# *Smed-dynA-1* is a planarian nervous system specific *dynamin 1* homolog required for normal locomotion

**DOI:** 10.1242/bio.20147583

**Published:** 2014-06-20

**Authors:** Jared A. Talbot, Ko W. Currie, Bret J. Pearson, Eva-Maria S. Collins

**Affiliations:** 1 Lewis-Sigler Institute for Integrative Genomics, Carl C. Icahn Laboratory, Princeton University, Princeton, NJ 08544, USA; 2 Program in Developmental and Stem Cell Biology, Hospital for Sick Children, Toronto, ON M5G 0A4, Canada; 3 Department of Molecular Genetics, University of Toronto, Toronto, ON M5S 1A8, Canada; 4 Ontario Institute for Cancer Research, Toronto, ON M5G 0A3, Canada; 5 Physics Department, University of California at San Diego, La Jolla, CA 92093, USA; 6 Division of Cell and Developmental Biology, University of California at San Diego, La Jolla, CA 92093, USA

**Keywords:** Dynamin, Planarian, Behavior, Locomotion

## Abstract

Dynamins are GTPases that are required for separation of vesicles from the plasma membrane and thus are key regulators of endocytosis in eukaryotic cells. This role for dynamin proteins is especially crucial for the proper function of neurons, where they ensure that synaptic vesicles and their neurotransmitter cargo are recycled in the presynaptic cell. Here we have characterized the dynamin protein family in the freshwater planarian *Schmidtea mediterranea* and showed that it possesses six dynamins with tissue specific expression profiles. Of these six planarian homologs, two are necessary for normal tissue homeostasis, and the loss of another, *Smed-dynA-1*, leads to an abnormal behavioral phenotype, which we have quantified using automated center of mass tracking. *Smed-dynA-1* is primarily expressed in the planarian nervous system and is a functional homolog of the mammalian Dynamin I. The distinct expression profiles of the six dynamin genes makes planarians an interesting new system to reveal novel dynamin functions, which may be determined by their differential tissue localization. The observed complexity of neurotransmitter regulation combined with the tools of quantitative behavioral assays as a functional readout for neuronal activity, renders planarians an ideal system for studying how the nervous system controls behavior.

## INTRODUCTION

Planarians are one of a few organisms that have the capability of regenerating an adult nervous system *de novo* upon injury. The molecular make-up of the planarian brain is slowly being described by *in situ* hybridization (ISH) and immunohistochemistry, which have revealed a surprising diversity of specialized neurons, including dopaminergic, octopaminergic, serotonergic, cholinergic, and GABAnergic neurons that form distinct neuronal networks (Cebrià, 2007; Nishimura et al., 2007a; Nishimura et al., 2007b; Cebrià, 2008; Nishimura et al., 2010; Currie and Pearson, 2013). Reliable communication at synapses of these neurons upon stimulation requires a certain number of vesicles that are “ready-to-go” to release a specific neurotransmitter. To achieve this prepared state, neurons continuously recycle the various proteins required for neurotransmitter release (Burgalossi et al., 2010).

Clathrin-mediated endocytosis (CME) is known to be one of the key pathways responsible for neurotransmitter recycling at presynaptic sites. Clathrin coated vesicles are budded from the membrane by Dynamins, large GTPases that are involved in early endocytosis as well as the regulation of actin dynamics for cell polarity, podosome formation, and cell migration (Goldstein et al., 1979; Ochoa et al., 2000; Sever, 2002; Chua et al., 2009; Nakayama et al., 2009). Mammals have three dynamin genes with distinctive expression patterns (Cook et al., 1996; Clark et al., 1997; Ferguson et al., 2007): Dynamin I is expressed primarily in the brain, Dynamin II is ubiquitous, and Dynamin III is expressed in the testis, lungs and brain. Each of these dynamins exists in at least four isoforms, which are generated by alternative splicing of mRNA (Urrutia et al., 1997).

Dynamin I, originally isolated from bovine brain (Shpetner and Vallee, 1989), has been extensively studied in *Drosophila melanogaster*, which has only one dynamin homolog, *shibire* (Chen et al., 1991). *Shibire* expression is strongest in the adult fly brain, but has also been detected in adult reproductive organs (Chen et al., 1992) and to lower levels in other tissues (Chen et al., 1992; Clark et al., 1997). Consistent with the strong neuronal expression, *Drosophila shibire* temperature sensitive mutants show a paralysis phenotype with impaired synaptic vesicle (SV) recycling when shifted to the non-permissive temperature (Grigliatti et al., 1973). Similarly, in the nematode *C. elegans*, a single dynamin gene, *dyn-1*, exists that is highly similar to the fly *shibire* gene and shows tissue specific expression in motor neurons, intestine, and pharyngeal muscle (Clark et al., 1997). A temperature sensitive mutation of *dyn-1* in *C. elegans* causes a locomotion phenotype and both, egg-laying and developmental defects, but full paralysis like in *Drosophila shibire* is not observed (Clark et al., 1997). At the restrictive temperature, the *dyn-1* mutation is embryonic lethal (Harris et al., 2001).

In this paper we present the first characterization of dynamin expression and function in the freshwater planarian *Schmidtea mediterranea*. We identified six planarian dynamin homologs, which we phylogenetically categorized into two clusters (A and B). We determined their expression profiles using ISH and antibody staining. Expression pattern analysis suggested partial redundancy between some dynamin homologs (*Smed-dynA-3* and *Smed-dynB-1* in the gut; *Smed-dynA-2*, *Smed-dynB-2* and *Smed-dynB-3* in the mesenchyme), with the exception of a single nervous system specific dynamin gene (*Smed-dynA-1*). Functional analysis using RNA interference (RNAi) (Sánchez Alvarado and Newmark, 1999) revealed that two planarian dynamin homologs were required for normal tissue homeostasis, while the loss of the nervous system specific dynamin, *Smed-dynA-1*, exclusively led to a characteristic locomotion phenotype, which we quantified using P-tracker automated center of mass tracking (Talbot and Schötz, 2011). Similar to the *C. elegans dyn-1* temperature-sensitive mutant, *Smed-dynA-1(RNAi)* worms never became fully paralyzed. They exhibited a combination of cilia- and musculature-driven locomotion, which in some cases resembled a serotonin-antagonist-induced locomotion phenotype two of us have previously described (Currie and Pearson, 2013). This finding strongly suggests that Smed-DYNA-1 is required for neurotransmitter recycling at presynaptic sites in planarians and serves as a functional homolog of the mammalian Dynamin I.

## RESULTS AND DISCUSSION

### Cloning and phylogenetic characterization of *dynamin* homologs in planarians

**Fig. 1.**
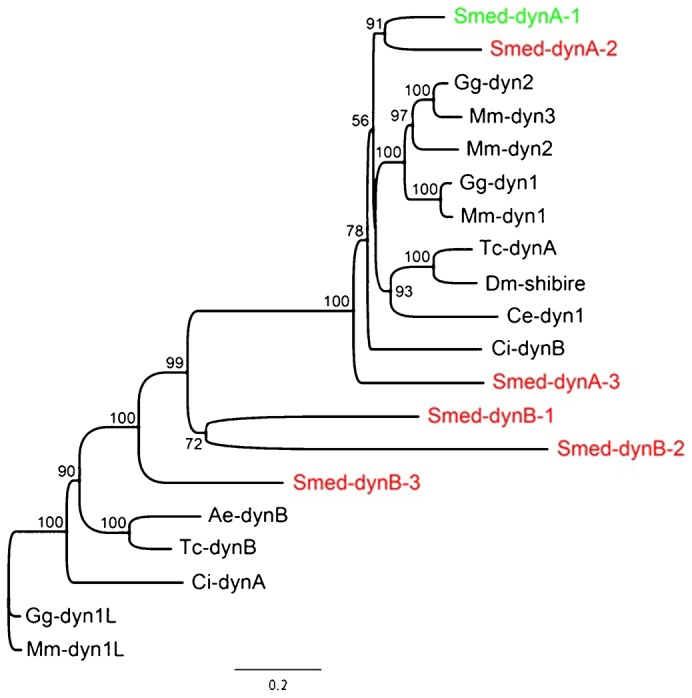
Bayesian phylogeny of Dynamin homologs with posterior probabilities >50% support shown at nodes. Planarians have 6 dynamins (red/green labels), 3 of which cluster with dynamin1–3 homologs from vertebrates and have been designated the A group. The 3 planarian Dynamins that cluster with the B group are more closely related to the Dynamin-1-like genes from vertebrates. The homolog in green represents the functional Dynamin I ortholog based on the experiments in this manuscript. Interestingly, the ascidian *Ciona intestinalis* and flour beetle *Tribolium castaneum* have homologs of both groups, suggesting that this state was ancestral and the B group was lost in *Drosophila* and *C. elegans*. Species in phylogeny: Mm – *Mus musculus* (mouse); Dm – *Drosophila melanogaster*; Tc – *Tribolium castaneum* (flour beetle); Smed – *Schmidtea mediterranea*; *Ce* – *C. elegans* (nematode); *Ci* – *Ciona intestinalis* (ascidian, cephalochordate); *Ae* – *Aedes aegypti* (mosquito); *Gg* – *Gallus gallus* (chicken). The scale indicates substitutions per site in branch length.

To find dynamin homologs in *S. mediterranea*, we performed extensive reciprocal BLAST analyses using fly and mouse dynamin protein sequences against the fully sequenced and assembled planarian genome and various available transcriptomes (Sánchez Alvarado et al., 2002; Sandmann et al., 2011; Labbé et al., 2012; Solana et al., 2012). From this, we identified a total of six predicted planarian dynamins, which we cloned using 3′ RACE. To phylogenetically categorize the planarian dynamins, the predicted protein sequences were aligned to other vertebrate and invertebrate dynamin sequences and subjected to Bayesian analysis. We found that 3 planarian dynamins clustered strongly with a group that includes fly Shibire and *C. elegans* Dyn-1, as well as Dynamins I–III in vertebrates (Fig. 1).

We have named this the A cluster and the genes are designated *Smed-dynA-1–3*. The B cluster contains some divergent gene duplicates, which are the dynamin-like gene from vertebrates and *Ciona*, and a gene duplicate from the flour beetle *Tribolium* (Fig. 1). Due to the close phylogenetic relationship with *Drosophila* Shibire and mammalian Dynamin-I, we hypothesized that the planarian dynamins belonging to the A cluster would be more likely to serve similar functions in the nervous system. Therefore, we next studied the expression patterns of the planarian dynamins to test whether any of them displayed expression in the nervous system.

### Planarian dynamins displayed tissue-specific expression patterns

**Fig. 2.**
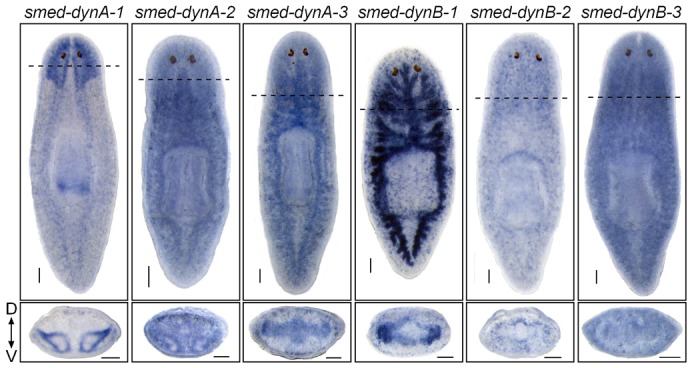
Expression patterns of dynamin family members. Whole-mount *in situ* hybridization (WISH) for *smed-dynA-1*, *smed-dynA-2*, *smed-dynA-3*, *smed-dynB-1*, *smed-dynB-2* and *smed-dynB-3*. All whole-mount stained images are dorsal views with the anterior of the worm at the top. Bottom panels are transverse sections of the worm at the site indicated by the dashed line, with the dorsal surface at the top. Scale bars: 100 µm.

Through the use of whole-mount *in situ* hybridization (WISH), we were able to observe that most planarian dynamin homologs display tissue-specific expression patterns. *Smed-dynA-3* and *Smed-dynB-1* transcripts were detected in the gut, while *Smed-dynA-2*, *Smed-dynB-2* and *Smed-dynB-3* displayed somewhat ubiquitous expression within tissues and associated mesenchyme. However, one dynamin (*Smed-dynA-1*) was strongly expressed throughout the planarian CNS and was detected in both the cephalic ganglia as well as the ventral nerve cords and the pharyngeal tip (Fig. 2). Therefore, we hypothesized that *Smed-dynA-1* is the functional homolog of *shibire* and the mammalian *dynamin I*, and fundamental for planarian nervous system function and behavior.

### Functional analysis of non-neuronal planarian dynamins

To determine the function of each dynamin, RNAi treatments were used to down-regulate gene expression (supplementary material Fig. S1). Knock down of *Smed-dynA-2* and *Smed-dynA-3* revealed each gene to be essential to survival and homeostasis. Both *Smed-dynA-2(RNAi)* and *Smed-dynA-3(RNAi)* animals showed disturbances in tissue homeostasis through the initial formation of dorsal lesions, eventually resulting in a lethal phenotype after only 2 RNAi feedings (supplementary material Fig. S2). Knockdowns of the *Smed-dynB* genes showed no noticeable phenotypes, even after more than 15 RNAi feedings, possibly due to redundancy with other dynamins with overlapping expression domains or incomplete knockdowns.

**Fig. 3.**
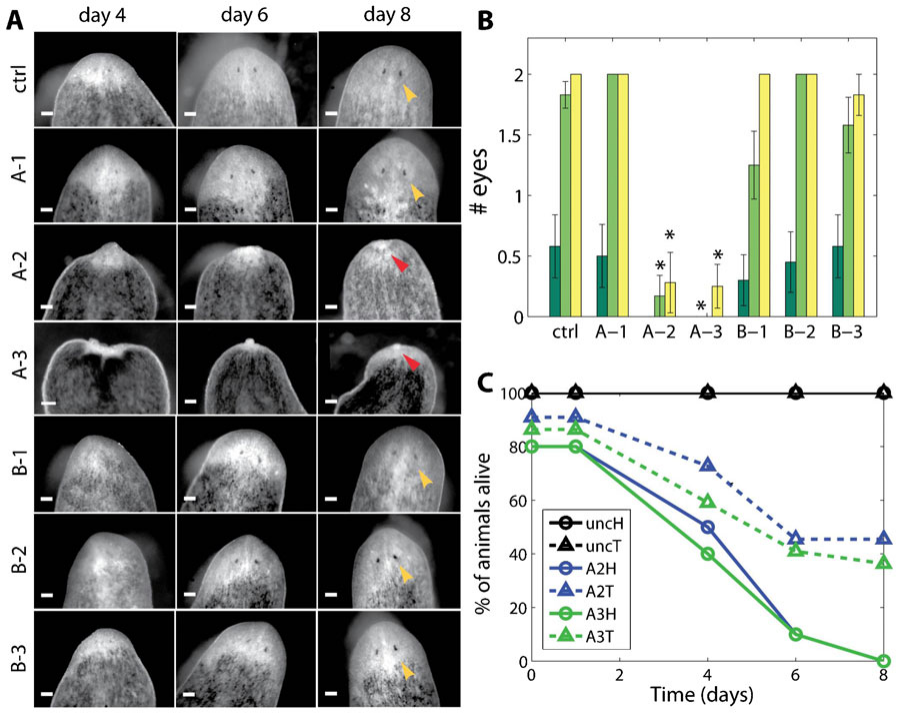
Two RNAi phenotypes lose their ability to regenerate. (A) Representative pictures of a planarian regeneration time course for each condition. Yellow arrows indicate 2 eyes at day 8, and red arrows indicate lack of eyes at day 8. (B) Quantification of eye appearance. Shown are the mean and s.e.m. of each population (n = 12; except for B-1 where n = 10) for days 4, 6, 8. Lack of error bars indicates that all worms in the group had 2 eyes. There is no significant difference in regeneration between the control and non-lethal RNAi populations, but *Smed-dynA-2(RNAi)* and *Smed-dynA-3(RNAi)* worms show statistically significant reduced regeneration. * corresponds to the 1% level. Missing bars for days 4 and 6 indicate that none of the worms in that group had eyes at this time point. (C) Death occurrence of *Smed-dynA-2(RNAi)* and *Smed-dynA-3(RNAi)* worms. Three worms were already dead at the time of amputation (day 0) and the majority of worms failed to regenerate and died within a week, with head fragments (circles) dying faster than tails (triangles). None of the *control(RNAi)* worms died during this time. Tail data are averaged over two experiments (n = 22), while head data were only recorded in one experiment (n = 10). Scale bars: 100 µm.

In addition, we performed a regeneration time course following RNAi to all planarian dynamin genes to ascertain whether any may be required for regeneration (for details, see Materials and Methods). Pictures of all RNAi worms were taken (n = 12 each, except for B-1 where n = 10) on days 1, 4, 6, and 8 after decapitation and eye appearance was quantified for days 4, 6, 8. The RNAi animals to the dynamin B homologs, which showed no intact homeostasis defects, also showed no significant difference in regeneration with controls (Fig. 3A,B). Furthermore, these worms continued asexual reproduction over the course of the RNAi treatments without any noticeable differences when compared to *control(RNAi)* worms. However, *Smed-dynA-2(RNAi)* and *Smed-dynA-3(RNAi)* animals that were fed twice before amputation displayed abnormal regeneration. A third of the worms displayed lesions at the time of amputation (35% for *Smed-dynA-2(RNAi)* and 37% for *Smed-dynA-3(RNAi)*). The majority of worms either died (55% for *Smed-dynA-2(RNAi)* and 64% for *Smed-dynA-3(RNAi)*) or did not complete head regeneration over the entire 8 day experiment as quantified by counting the number of eyes per worm (Fig. 3B). Temporal dynamics of death occurrence for *Smed-dynA-2(RNAi)* and *Smed-dynA-3(RNAi)* animals was quantified and is shown in Fig. 3C. By day 4, half of the head fragments and a third of the tail fragments had already died, and by day 8 all heads had vanished and only about 50% of tails were still alive (Fig. 3C).

Epidermal lesions are often a hallmark of stem cell defects in planarians (Reddien et al., 2005b; Labbé et al., 2012). Therefore, we assayed the stem cell population, using the marker *smedwi-1*, in *smed-dynA-2(RNAi)* and *smed-dynA-3(RNAi)* worms that displayed epidermal lesions (14 days after the second RNAi feeding). However, based on the WISH analyses for *smedwi-1* expression, no apparent defects within the stem cell population were detected (supplementary material Fig. S2). This suggested that these dynamins may play a role in epithelial integrity as opposed to having a stem cell-based tissue turnover function.

For the remainder of this paper we turn our attention to the single nervous system specific dynamin, *Smed-dynA-1*, which was the best candidate for a *dynamin-I* homolog that could link dynamin mediated endocytosis to animal behavior.

### SMED-DYNA-1 is localized at synapses and is a functional homolog of Dynamin I in flies (*shibire*) and vertebrates

**Fig. 4.**
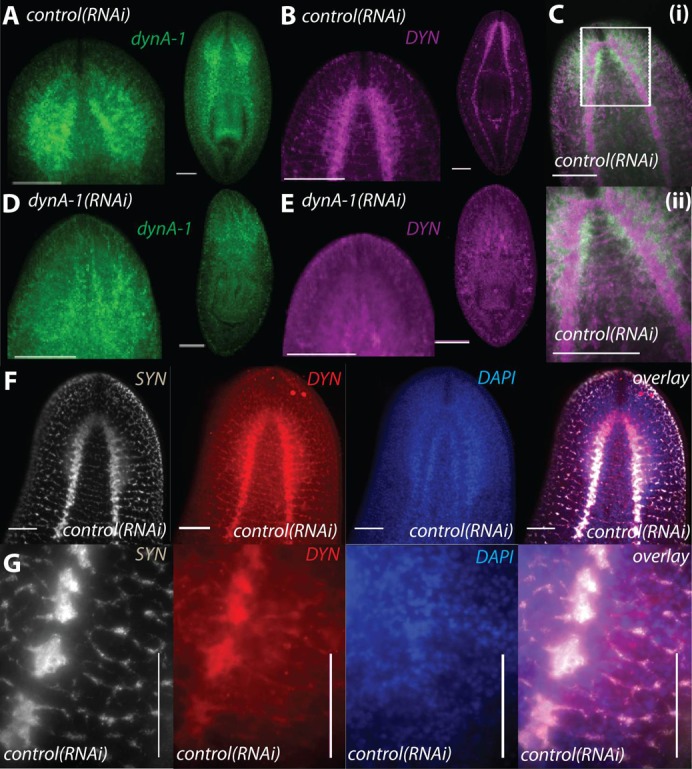
*Smed-dynA-1* RNA and SMED-DYNA-1 protein are specifically localized to the brain. (A) Fluorescent *in situ* hybridization (FISH) of *Smed-dynA-1* RNA and (B) antibody staining of SMED-DYNA-1 protein using a commercial anti-DYNAMIN-1 antibody in *control(RNAi)* worms. (C) While the mRNA is localized in the cell bodies, the protein is localized at the synapse. (Cii) A higher magnification image of the area outlined in panel Ci. (D,E) FISH of *Smed-dynA-1* RNA (D) and anti-DYNAMIN-1 (E) staining of *Smed-dynA-1(RNAi)* worms. The lack of expression shows the specificity of the antibody and effectiveness of RNAi treatment. (F,G) Double antibody staining of anti-SYNAPSIN (SYN), anti-DYNAMIN-1(DYN) and DAPI in the brain at 10× (F) and nerve cords at 40× magnification (G) shows co-localization at the synapse. Scale bars: 100 µm.

Although *Smed-dynA-1* was clearly localized to the CNS of planarians (Fig. 2, Fig. 4A), we predicted that the protein product of this gene should be localized to synaptic regions if it was involved in vesicle recycling. Using an antibody made to rat DYNAMIN I, we found that indeed, specific labeling to the protein occurs in the neuropil of the brain where axon tracts and synapses are most dense (Fig. 4B,C). We confirmed that anti-DYNAMIN I specifically labels SMED-DYNA-1 protein by staining *Smed-dynA-1(RNAi)* worms (5 days after 12 RNAi feedings) with this rat antibody, which showed little to no discernible staining compared to a robust staining of the *control(RNAi)* worms (Fig. 4E). In addition, strong co-localization was observed between SMED-DYNA-1 and the established synaptic protein, SYNAPSIN, by double immunolabeling experiments, adding further support that SMED-DYNA-1 is localized to synapses in the planarian nervous system (Fig. 4F,G).

Interestingly, in *Drosophila*, labeling with a rat anti-DYNAMIN I antibody was also specific to the brain (Chen et al., 1991; Chen et al., 1992), further supporting the hypothesis that the planarian homolog may have the canonical neural function of fly *shibire* and mammalian *dynamin I*. Following RNAi of *Smed-dynA-1*, we observed that animals were fully viable and did not show any regeneration or reproduction defects (Fig. 3). We did, however, observe that *Smed-dynA-1(RNAi)* worms displayed qualitatively aberrant behavior and slower, uncoordinated locomotion when compared to wild-type and *control(RNAi)* worms (supplementary material Movies 1–3). We next used worm tracking software and statistical methods to further quantify the locomotion defects of the *dynA-1(RNAi)* phenotype.

### Quantitative behavioral analysis of the *Smed-dynA-1(RNAi)* phenotype

**Fig. 5.**
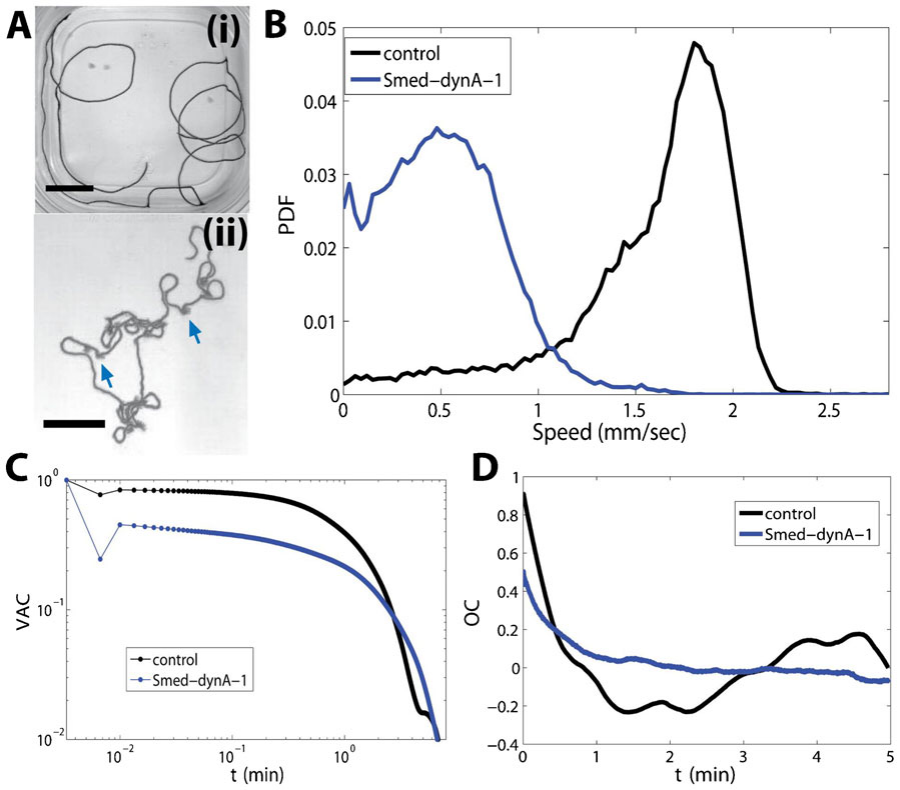
Quantification of *Smed-dynA-1(RNAi)* behavior. (A) Representative minimum intensity projection of (i) a *control(RNAi)* planarian track and (ii) a *Smed-dynA-1(RNAi)* planarian track. (B) The speed probability density function (PDF) shows that *Smed-dynA-1(RNAi)* worms move at about 3.5 times slower than *control(RNAi)* worms. (C) Velocity autocorrelation (VAC) versus timelag (τ) on log–log scale. The control group moves straighter and thus has initially a higher VAC, but then loses the correlation faster due to the higher velocity, causing worms to hit the container boundaries faster. The rapid dip at the second datapoint is due to noise. (D) Average orientation correlation (OC; not normalized) versus timelag (τ). The *Smed-dynA-1(RNAi)* curve is flat due to their lower migration speed and lacks the anti-correlated state observed in the *control(RNAi)* population because these worms hardly move along the container boundaries, in contrast to the *control(RNAi)* worms. n = 15 planarians of each type. Data were taken at 5 frames per second, 3000 frames for each worm. Scale bars: 2 cm.

Based on the exclusive nervous system specific expression profile of SMED-DYNA-1, it was expected that RNAi treatment would cause a behavioral phenotype, similar to observations in *Drosophila* or *C. elegans*. Qualitatively these worms displayed erratic and uncoordinated locomotion when compared to *control(RNAi)* worms (representative tracks are shown in Fig. 5A; see also supplementary material Movies 1–3). Using center of mass (COM) tracking, we were able to quantitatively characterize their behavior (Talbot and Schötz, 2011). The first observation was the significant difference in speed: *Smed-dynA-1(RNAi)* worms displayed a nearly 3.5-fold decreased speed when compared to *control(RNAi)* worms (mode of 0.48 mm/sec versus 1.80 mm/sec; Fig. 5B). What was also noteworthy was the skew of the speed distribution to zero speeds for *Smed-dynA-1(RNAi)* worms, which was due to frequent stopping, turning and head wiggling, as can be seen from the representative track (Fig. 5A). The latter caused local fan-like widening of the trajectory; two instances are indicated by the arrows in Fig. 5Aii. This difference in speed was also reflected in the velocity autocorrelation (VAC; Fig. 5C; Table 1; for details on the calculation, see Talbot and Schötz, 2011), which allows us to determine the directedness of motion. If a worm would move straight with constant velocity, the VAC in Fig. 5B would show a horizontal line. However, due to stops, turns and movements, which cause the worm to change speed and/or direction, the VAC drops over time. Using an exponential fit, we can extract a time scale, the persistence time, which is the time in which worms move in roughly the same direction. Because of the lower speed, *Smed-dynA-1(RNAi)* worms display a longer persistence time (112±2 sec versus 73±3 sec; mean ± s.e.m.; Fig. 5C; Table 1). The persistence time of *control(RNAi)* worms is largely set by the size of the container – because they move fast, they reach the boundary quickly, upon which their direction of motion changes, causing them to lose correlation. One can quantitatively see this by determining a “persistence length” of motion, multiplying the worms' average velocity with the persistence time, resulting in 5.4 cm for *Smed-dynA-1(RNAi)* worms and about 13.1 cm for *control(RNAi)* planarians, which roughly corresponds to the size of the dish (length 14 cm). The persistence time obtained for the *control(RNAi)* worms is a bit higher than our previously published results for wild-type worms (61±3 sec; mean ± s.e.m.) (Talbot and Schötz, 2011). This is due to the slightly reduced speed (our wild-type worm sample had a mode of 1.92 mm), which, however, lies within the expected inter-worm variability and is not a sign of a locomotion phenotype (Talbot and Schötz, 2011).

**Table 1.**
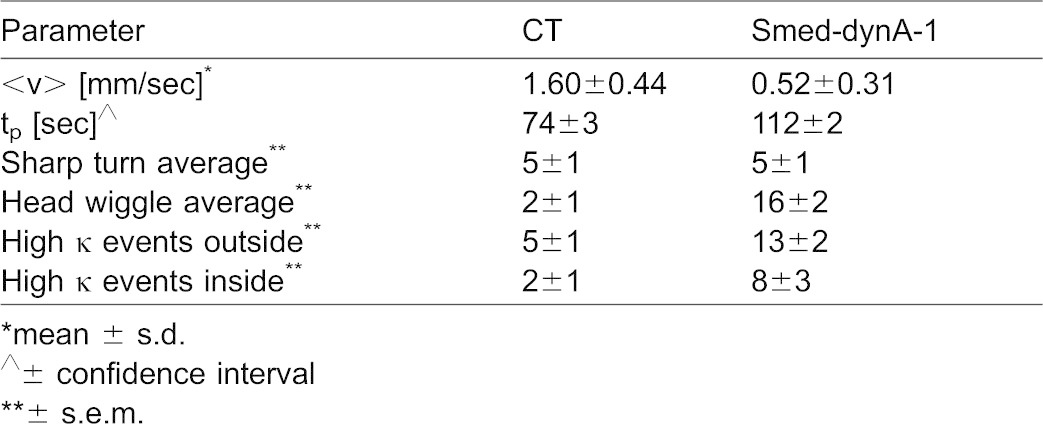
Summary of parameters

In contrast to the long straight runs of *control(RNAi)* worms, *Smed-dynA-1(RNAi)* planarians displayed an increased turning rate, crossed their own tracks with greater frequency, and often never reached the boundary of the dish (Fig. 5A). A quantitative measure for this lack of spatial exploration is the angular component of the VAC, which we referred to previously as orientation correlations (OC; for details, see Talbot and Schötz, 2011). As expected from their tracks, *Smed-dynA-1(RNAi)* worms showed low initial correlation when compared to controls, and this correlation quickly dissipated to zero, indicating the limited spatial exploration by *Smed-dynA-1(RNAi)* worms (Fig. 5D). In contrast, the OC for *control(RNAi)* worms started out high and then switched between positive and negative values, a consequence of circular motion around the dish (see also track in Fig. 5Ai).

Besides frequent turning and limited spatial exploration, the other characteristic feature of *Smed-dynA-1(RNAi)* worm behavior is the higher rate of head motion and re-orientation.

**Fig. 6.**
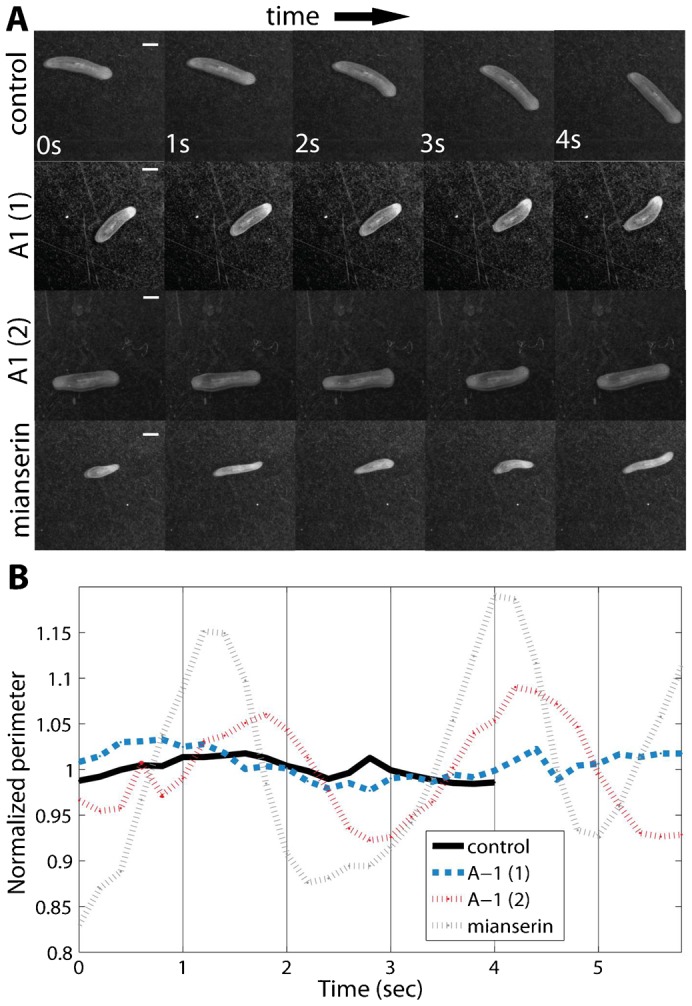
*Smed-dynA-1(RNAi)* worms display a mixture of cilia and musculature-driven locomotion. (A) Representative images of worm locomotion of (from top to bottom) *control(RNAi)*, *Smed-dynA-1(RNAi)* and mianserin treated wild-type planarians, taken at 1 sec intervals. Time progresses from left to right. *Smed-dynA-1(RNAi)* planarians display a mix of cilia-driven (dynA-1 (1)) and musculature-driven (dynA-1 (2)) locomotion. The latter strongly resembles mianserin-induced inchworm-like crawling of a wild-type planarian. (B) Normalized worm perimeter as a function of time. Inchworm-like crawling manifests itself in periodic fluctuations of the body perimeter as the planarian contracts and extends. The representative pictures in panel A correspond to the worm shapes at 0, 1, 2, 3 and 4 sec in this plot. The control data are slightly shorter since the worm left the field of view faster due to the increased speed when compared to the other samples. Scale bars: 1 mm.

As shown previously for drug-induced phenotypes (Talbot and Schötz, 2011), head wiggles and turns can be quantified by calculating the instantaneous speed (s) and curvature (κ), versus *distance traveled* (instead of versus time), to quantify the number of sharp turns (κ>1 mm^−1^ and s<0.1 mm/s) and head wiggles (κ>1 mm^−1^ and s>0.1 mm/s). While the average number of sharp turns per run was similar among the two groups, the tendency to head wiggle was roughly eight times greater in *Smed-dynA-1(RNAi)* worms (Table 1). As we have shown previously (Talbot and Schötz, 2011), interactions with the boundary of the container often result in turns or head movement (high curvature events). Interestingly, in the case of *Smed-dynA-1(RNAi)* worms, we found similar numbers of high curvature events in the interior of the container (“inside”) and the outer areas of the container, where interactions with the boundary were possible (“outside”). This similarity indicated that *Smed-dynA-1(RNAi)* worms intrinsically have a tendency to curve back on themselves, whereas *control(RNAi)* worms, wild-type worms and the two drug-induced locomotion phenotypes, which we previously characterized, displayed high curvature events primarily as a consequence of interactions with the boundary (Table 1) (Talbot and Schötz, 2011). Furthermore, a distinct characteristic of this RNAi behavioral phenotype is that the animals eventually display a combination of cilia- and musculature-driven locomotion (Fig. 6; supplementary material Movies 1–4). Cilia-driven locomotion allows planarians to glide smoothly without body deformation (Fig. 6; supplementary material Movie 1), whereas musculature-driven locomotion causes worms to wiggle their body substantially (Fig. 6; supplementary material Movie 2) and to display distinctive inchworm-like crawling (Fig. 6; supplementary material Movie 3).

This kind of musculature-driven motion strongly resembles a serotonin antagonist (mianserin) induced behavioral phenotype, which we have previously described (Currie and Pearson, 2013) (Fig. 6B; supplementary material Movie 4) and suggests that *Smed-dynA-1* function is required for serotonin recycling and cilia function.

In summary, these results demonstrate that *Smed-dynA-1(RNAi)* worms displayed a distinctive behavioral phenotype, which was characterized by slow locomotion resulting from a mix of cilia and musculature driven propulsion, decreased spatial exploration due to curved trajectories, and increased body movements, particularly of the head. This increased head activity may be an overcompensation mechanism of the animal for the lack of dynamin to help with reorientation away from negative stimuli (light, open spaces).

### Conclusions

The planarian *S. mediterranea* has six dynamins, which display tissue specific expression profiles; two of these are essential for tissue homeostasis and one for normal locomotion. The existence of multiple dynamins and the specificity of a mammalian anti-Dynamin I antibody to the planarian nervous system specific SMED-DYNA-1 protein suggests that the planarian dynamin *Smed-dynA-1* functions as the *dynamin I* homolog. The richness of the dynamin gene family in planarians makes them an interesting new invertebrate system to test novel dynamin functions in and outside the nervous system, and sets them apart from classical models such as *C. elegans* and *D. melanogaster*.

We have previously shown the capacities of automated center of mass tracking for a quantitative distinction of drug-induced locomotion phenotypes (Talbot and Schötz, 2011) but not for screening RNAi worms. Here we further show the value of our tracking method to quantify the *Smed-dynA-1(RNAi)* locomotion phenotype. What is particularly striking about this RNAi behavioral phenotype is a combination of cilia- and musculature-driven locomotion, which resembled a serotonin antagonist (mianserin) induced phenotype (Currie and Pearson, 2013). This suggests that *Smed-dynA-1* function is required for serotonin recycling and cilia function.

As is the case for the *C. elegans dyn-1* mutant, *Smed-dynA-1(RNAi)* worms do not reach full paralysis. There are several possible explanations for this finding: First, the RNAi may not fully penetrate, leading to a hypomorphic condition of partial locomotion. Second, redundant mechanisms may exist that compensate for the loss of *smed-dynA-1* function in SV recycling. One possibility could involve increased fluid-phase endocytosis as is observed in mammalian cell culture systems (Guha et al., 2003). Alternatively, planarian synaptic membranes may rely on the activity of fusion pores, which are thought to only transiently fuse with the presynaptic membrane for neurotransmitter release before reconfiguring for a new round of exocytosis (“kiss-and-run”) (Urrutia et al., 1997; Fernández-Peruchena et al., 2005). Third, different types of neurons may be differently affected by the smed-dynA-1 knockdown, thus leading to aberrant behavior but not complete paralysis. For example, in *dynamin-I* knockout mice, a strong heterogeneity in vesicle recycling modes as a function of synapse type (inhibitory versus excitatory) and activity state has been reported (Hayashi et al., 2008). A similar heterogeneity may exist in planarians and thus cause this partial behavioral phenotype. The exploration of these alternative endocytosis mechanisms relative to CME at synaptic sites in the planarian will be an interesting avenue for future research. Based on the complexity of their nervous system and regulation of neuronal activity, and their amenability for quantitative behavioral assays to test for neuronal function, planarians have the potential of becoming an important model for elucidating the link between neuronal activity and behavior.

## MATERIALS AND METHODS

### Worm maintenance and RNAi treatment

Asexual clones CIW4 *S. mediterranea* were used for all experiments. Worms were stored at 20°C in the dark in planarian water as previously described (Talbot and Schötz, 2011), except during feeding, cleaning, and data acquisition. For some RNAi experiments, the beef liver homogenate was mixed with dsRNA-expressing bacteria and directly added to the worms (Reddien et al., 2005b). For other experiments, RNAi knockdowns were generated by feeding *in vitro* transcribed dsRNA (Collins et al., 2010). As a control RNAi group, worms were fed *unc22* double-stranded RNA. *Unc22* encodes a *C. elegans* gene that is absent in planarians (Reddien et al., 2005a). *Smed-dynA-1(RNAi)* and *unc22 control(RNAi)* worms were fed at least 12× over the course of several weeks before experiments. The other dynamin RNAi worms were fed until death occurred (*Smed-dynA-3*, *Smed-dynA-2*) or until the experiment was stopped without having obtained a significant phenotype (*Smed-dynB-1*, *Smed-dynB-2*, *Smed-dynB-3*). Some of the *Smed-dynA-1(RNAi)* and *control(RNAi)* worms were imaged and fixed for *in situ* hybridization or antibody staining, others were used for the locomotion experiments.

### Molecular cloning and sequence analysis

The six *S. mediterranea dynamin* homologs were cloned from extracted planarian total RNA, reverse transcribed into cDNA, and 3′-RACE cloned as previously described (Pearson and Sánchez Alvarado, 2010). The Smed-dynA-1 PCR product (887 bp) was TA-cloned into PCR4-TOPO (Invitrogen) for *in situ* hybridization or into the pPR244 or pT4P vector for RNAi (kindly provided by A. Sànchez Alvarado). The other dynamins were cloned into pT4P for RNAi and riboprobes as previously described (Pearson et al., 2009). Cloned ORFs were then converted to predicted proteins and subjected to Maximum Likelihood and Bayesian phylogenetic analyses. Protein sequences used in phylogenies were obtained from the NCBI Entrez protein database. The program Geneious (http://www.geneious.com) was used with the MUSCLE alignment plugin and two tree building plugins for Geneious were used as independent analyses. Both Maximum Likelihood and Bayesian analyses were performed with the following settings: (Maximum Likelihood – 100 bootstrap replicates, WAG substitution model, estimated distances. Bayesian – 1 million replicates, WAG substitution model, 4 heated chains, 25% burnin, subsample frequency of 1000). Consensus trees were saved through Geneious as .jpgs, which were then manipulated in Adobe Photoshop. A FASTA file of all protein and nucleotide sequences as well as alignments can be provided upon request.

### *In situ* hybridization and antibody staining

Whole-mount and fluorescent *in situ* hybridizations (WISH and FISH) were performed as previously described (Pearson et al., 2009). For both methods, planarians were collected 7–10 days following feeding and treated 30 sec–1 min in 2% HCl in Phosphate Buffered Saline (PBS), followed by a 10 min fixation on a nutator in 4% (para-) formaldehyde at room temperature (RT). Worms were bleached in 6% hydrogen peroxide in Methanol and then stored in methanol at −20°C until used. BCIP/NBT were used for WISH and NHS-fluorescein or NHS-rhodamine (1:1000), synthesized following a protocol by Lance Davidson (Pearson et al., 2009), was used for FISH. Immunohistochemistry samples where incubated for 4–5 hours at RT in a blocking solution containing 89% PBSTT (PBS; 0.1% Tween-20; 0.3% Triton X-100), 10% fetal calf serum (FCS), and 1% Dimethyl sulfoxide (DMSO). Samples were then incubated at RT for 4 hours in a commercial mouse anti-rat Dynamin I antibody (BD Biosciences, cat. no. 610245) diluted 1:500 in blocking solution. The signal was detected using an Alexa Fluor 546 rabbit anti-mouse secondary antibody (1:1000; Invitrogen, cat. no. A-11060). Planarians were mounted in custom made tunnel slides and imaged on an Olympus IX81 DSU microscope (Olympus, Center Valley, PA) using Slidebook software (Intelligent Imaging Innovations, Inc.). Tunnel slides consisted of the specimen being placed on coverglass between double sticky tape, covered with a square coverglass, and sealed with silicone grease or nail polish. For double-antibody staining, an anti-mouse HRP antibody (1:1000; Enzo Life Sciences) was used as secondary antibody, followed by a NHS-fluorescein tyramide reaction. The antibody was stripped off by incubating for 10 min in 0.1 M glycine-HCl, pH 2.2, and 0.1% Triton X-100. Samples were subsequently incubated in a mouse anti-synapsin (SYNORF1) antibody (1:500; Developmental Studies Hybridoma Bank), followed by another incubation in anti-mouse HRP and a NHS-TRITC tyramide reaction.

### Regeneration assay

RNAi treated planarians were decapitated 5 days after twelve feedings (12fd5) for *Smed-dyn-1* (n = 12) and the *control* (n = 12), 8fd5 for the other non-lethal (n = 12; for B-1 n = 10), and 2fd5 for the lethal phenotypes (at the onset of skin lesions; n = 12) were decapitated and moved into individual petri dishes. They were stored at 20°C in the dark in planarian water, except during imaging. Worms were not fed during the regeneration period, but they were all transferred to fresh containers on day 4 of the experiment. They were individually imaged on a Leica MZ16FA stereo microscope on days 1, 4, 6, and 8. The number of eyes was quantified on days 4, 6, and 8 by visual inspection and manual counting. A χ^2^ statistical analysis was performed to test for differences among the groups for days 6 and 8. For the lethal phenotypes, we performed an additional round of regeneration assays, 2fd3 (n = 10) to quantify phenotype penetrance. The *C. elegans* gene, *unc22*, was used as a control RNAi construct (n = 10).

### Quantitative behavioral analysis

Quantitative behavioral assays were carried out as described previously (Talbot and Schötz, 2011). In brief, worms were imaged using LabVIEW software (National Instruments, version 8.5), a A601f Basler camera (Basler, Germany) and simple Gauss lens (Edmund Optics, NT55-326), mounted on a ring stand. Frames were acquired at 5 frames per second for 10 min for each worm. n = 15 worms were used for each group. Center of mass tracking was accomplished using our P-tracker software. Data analysis in MATLAB was carried out as previously described (Talbot and Schötz, 2011).

### Perimeter quantification

Image sequences were obtained by recording planarians moving freely in a petri dish using a Leica MZ16FA stereo microscope, equipped with a Basler A601f camera and a custom MATLAB image acquisition script. Images were analyzed and the worm perimeter extracted using standard image analysis methods and the analyze particle feature in Image J 1.48 (NIH). Perimeter values were normalized by the mean perimeter for each group and the results plotted in MATLAB.

## Supplementary Material

Supplementary Material
