# Supplementary material for: Smed-dynA-1 is a planarian nervous system specific dynamin 1 homolog required for normal locomotion
Source: Biol Open. 2014 Jun 20;3(7):627–34. doi: 10.1242/bio.20147583 (PMC4154299; doi:10.1242/bio.20147583)
Supplement: Supplementary Material [file supp_3_7_627__index.html]

Smed-dynA-1 is a planarian nervous system specific dynamin 1 homolog required for normal locomotion — Smed-dynA-1 is a planarian nervous system specific dynamin 1 homolog required for normal locomotion — Supplementary Material 

# *Smed-dynA-1* is a planarian nervous system specific *dynamin 1* homolog required for normal locomotion

## bio.20147583 Supplementary Material

**Files in this Data Supplement:**

- Supplementary Material - Jared A. Talbot et al. doi: 10.1242/bio.20147583
- Movie 1 - **Movie 1. The locomotion of a *control(RNAi)* planarian acquired at 5 frames/sec**. Real time is shown in top left corner. Scale bar: 1 mm.
- Movie 2 - **Movie 2. The locomotion of a *smed-dynA-1(RNAi)* planarian acquired at 5 frames/sec**. *smed-dynA-1(RNAi)* planarians display a mix of cilia-driven (this movie) and musculature-driven (supplementary material Movie 3) locomotion. Real time is shown in top left corner. Scale bar: 1 mm.
- Movie 3 - **Movie 3. The locomotion of a *smed-dynA-1(RNAi)* planarian acquired at 5 frames/sec**. *smed-dynA-1(RNAi)* planarians display a mix of cilia-driven (supplementary material Movie 2) and musculature-driven (this movie) locomotion. Real time is shown in top left corner. Scale bar: 1 mm.
- Movie 4 - **Movie 4. The musculature driven locomotion of a wild-type planarian treated locally with 200 µL of 200 µM mianserin and acquired at 5 frames/sec**. Real time is shown in top left corner. Scale bar: 1 mm.
