# Supplementary material for: Smed-dynA-1 is a planarian nervous system specific dynamin 1 homolog required for normal locomotion
Source: Biol Open. 2014 Jun 20;3(7):627–34. doi: 10.1242/bio.20147583 (PMC4154299; doi:10.1242/bio.20147583)
Supplement: Supplementary Material [file supp_bio.20147583_bio.20147583-s1.pdf]

Biology Open (2014) 000, 1–8 doi:10.1242/bio.20147583

Supplementary Material
Jared A. Talbot et al. doi: 10.1242/bio.20147583

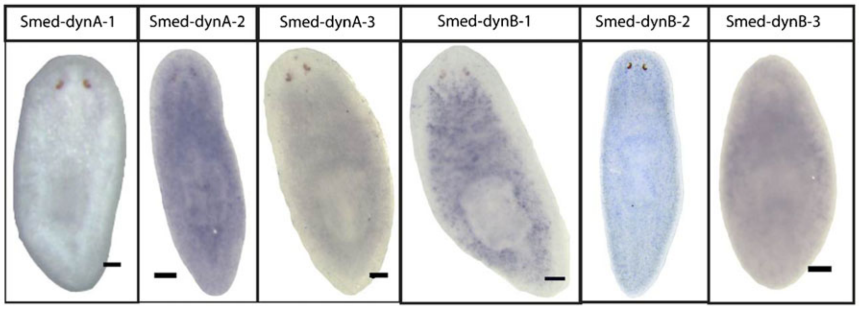

Fig. S1. ***In situ*** **hybridization confirms effectiveness of RNAi treatment.** WISH images for dynamins *smed-dynA-1(RNAi)*, *smed-dynA-2(RNAi)*, *smed-dynA-3(RNAi)*, *smed-dynB-1(RNAi)*, *smed-dynB-2(RNAi)* and *smed-dynB-3(RNAi)* after two (lethal phenotypes; 2fd7 and twelve RNAi feedings (12fd7). Scale bars: 100 µm.

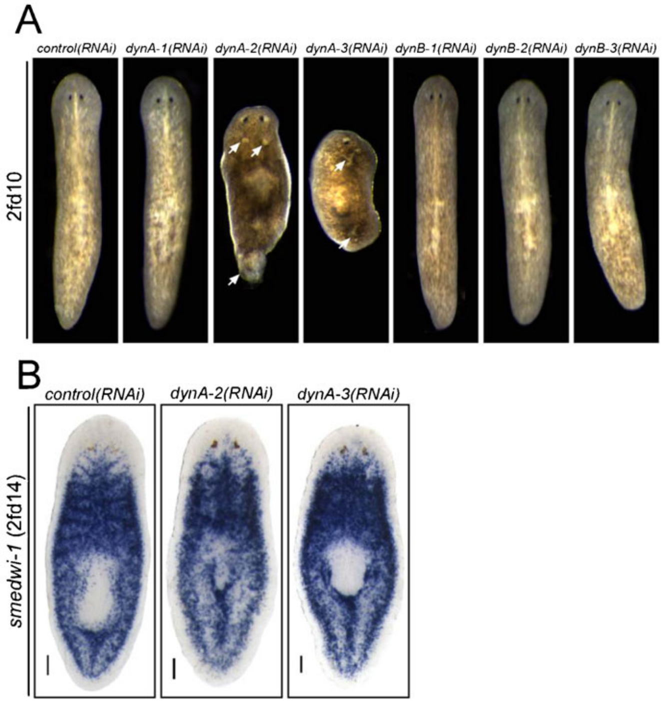

Fig. S2. Analysis of *dynamin(RNAi)* worms. (A) Tissue homeostasis defects are observed in *smed-dynA-2(RNAi)* and *smed-dynA-3(RNAi)* worms as lesions on the outer epithelium (white arrows). All worms were subjected to two RNAi feedings and live images were taken 10 days later (2fd10). (B) WISH images for the stem cell marker *smedwi-1* in *smed-dynA-2(RNAi)* and *smed-dynA-3(RNAi)* worms. Worms were examined 14 days after the second RNAi feeding (2fd14). All images are dorsal views with the anterior of the worm facing up. Scale bars: 100 µm.

Biology Open (2014) 000, 1–8 doi:10.1242/bio.20147583

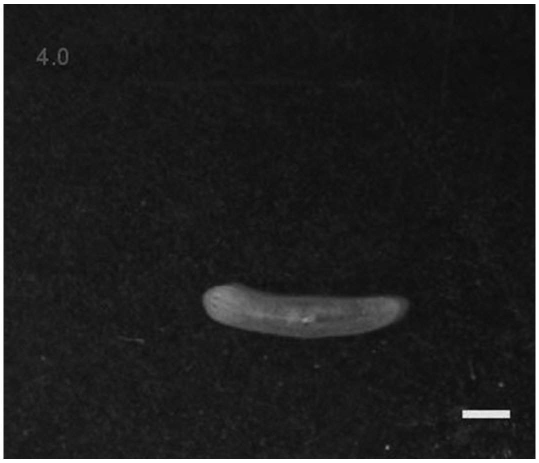

Movie 1. The locomotion of a *control(RNAi)* planarian acquired at 5 frames/sec. Real time is shown in top left corner. Scale bar: 1 mm.

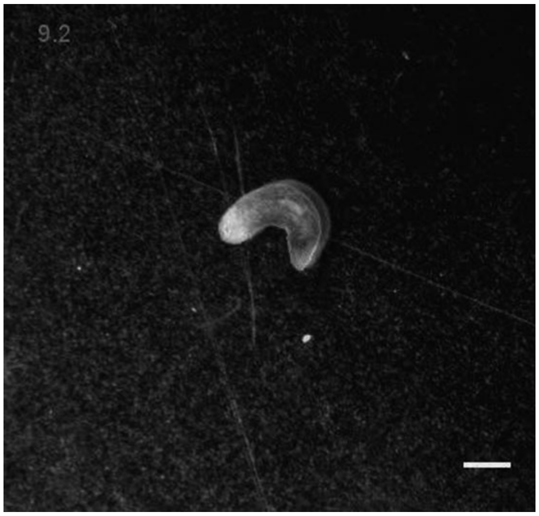

Movie 2. The locomotion of a *smed-dynA-1(RNAi)* planarian acquired at 5 frames/sec. *smed-dynA-1(RNAi)* planarians display a mix of cilia-driven (this movie) and musculature-driven (supplementary material Movie 3) locomotion. Real time is shown in top left corner. Scale bar: 1 mm.

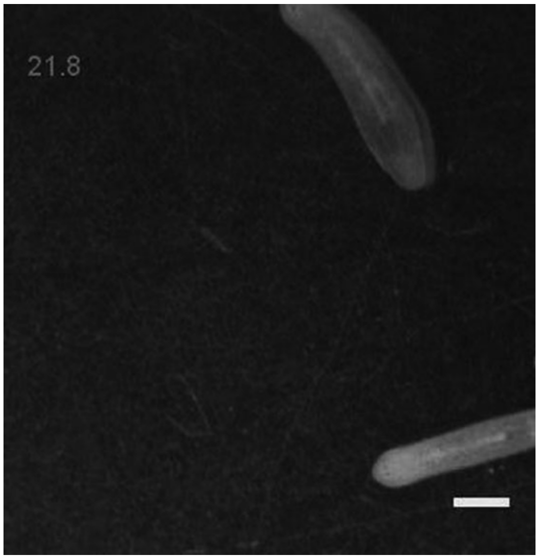

Movie 3. The locomotion of a *smed-dynA-1(RNAi)* planarian acquired at 5 frames/sec. *smed-dynA-1(RNAi)* planarians display a mix of cilia-driven (supplementary material Movie 2) and musculature-driven (this movie) locomotion. Real time is shown in top left corner. Scale bar: 1 mm.

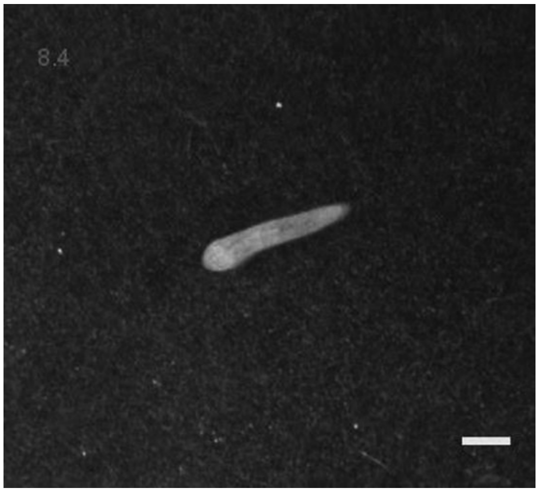

Movie 4. The musculature driven locomotion of a wild-type planarian treated locally with 200 μL of 200 μM mianserin and acquired at 5 frames/sec. Real time is shown in top left corner. Scale bar: 1 mm.
